# Knowledge and perceptions of nicotine, smoking cessation and electronic nicotine delivery systems among physicians and pharmacists in a Swiss hospital group

**DOI:** 10.18332/tid/204839

**Published:** 2025-07-24

**Authors:** Samuel E. Christen, Elisabetta Scanniello, Felix Hammann, Carla Meyer-Massetti, Reto Auer, Jean-François Etter, Evangelia Liakoni

**Affiliations:** 1 Clinical Pharmacology and Toxicology, Department of General Internal Medicine, Inselspital, Bern University Hospital, University of Bern, Bern, Switzerland; 2 Graduate School for Health Sciences, University of Bern, Bern, Switzerland; 3 Institute of Primary Health Care (BIHAM), University of Bern, Bern, Switzerland; 4 Center for Primary Care and Public Health (Unisanté), University of Lausanne, Lausanne, Switzerland; 5 Institute of Global Health, Faculty of Medicine, University of Geneva, Geneva, Switzerland

**Keywords:** nicotine, electronic nicotine delivery systems, e-cigarettes, survey, smoking cessation

## Abstract

**INTRODUCTION:**

Despite the important role that healthcare professionals play in smoking cessation strategies, recent reports from several countries show misperceptions about nicotine, pharmacotherapy for smoking cessation and novel nicotine products, but little is known about such knowledge gaps among healthcare professionals in Switzerland.

**METHODS:**

This study involved a cross-sectional anonymous survey. Physicians and pharmacists from a large hospital group in Switzerland were invited in 2023 by e-mail to participate. The survey covered nicotine, smoking cessation, and knowledge of electronic nicotine delivery systems (ENDS).

**RESULTS:**

Of the 2035 healthcare professionals contacted, 279 responded to the survey (14%). Fifty-three percent of participants identified as women, 69% were in the age group of ≤40 years, 77% were never smokers, and 85% saw patients daily. The majority (76%) agreed that nicotine is the main substance in tobacco responsible for addiction, while 73% and 69% disagreed that nicotine on its own causes cancer or chronic obstructive pulmonary disease (COPD), respectively. Most participants (n=128; 63%) opposed the recommendation of e-cigarettes as a smoking cessation aid, although e-cigarettes were considered less harmful than combustible cigarettes, both for users and bystanders. Nevertheless, 64% considered them to be equally or more problematic for public health than tobacco cigarettes.

**CONCLUSIONS:**

This survey highlights knowledge gaps and misperceptions about nicotine and smoking cessation products among healthcare professionals in a large hospital group in Switzerland. Respondents appeared to have a relative accurate understanding regarding most of the direct effects of nicotine. However, uncertainties were noted in relation to newer products such as e-cigarettes. Future research should extend to other healthcare professionals and assess the impact of targeted training on knowledge and clinical practice.

## INTRODUCTION

Nicotine addiction drives smoking, but it is the toxic, carcinogenic, and airway irritating combustion products that are primarily responsible for the deleterious effects of smoking, such as chronic obstructive pulmonary disease (COPD), cardiovascular disease and cancer^1^. First-line therapies for smoking cessation include nicotine replacement therapy (NRT), the nicotinic α4β2 receptor partial agonist varenicline (not available in Switzerland since 2021, when the manufacturer halted production due to nitrosamine impurities^2^) and the dopamine and norepinephrine reuptake inhibitor bupropion. These treatments offer valuable support to smokers who want to quit, but success rates remain low (<30% after one year)^3^. In Switzerland, bupropion must be prescribed by physicians, varenicline can be dispensed by pharmacists with appropriate documentation and therapeutic follow-up, and NRT products are available over the counter.

To increase smoking cessation rates, routine screening and guidance by medical staff are crucial. However, reports from other countries show that healthcare professionals have misconceptions about nicotine, smoking cessation pharmacotherapy and/or ENDS. To name a few examples, in a survey of Greek healthcare professionals, more than 30% believed that NRT products were as or more addictive than tobacco cigarettes^4^. A survey of European healthcare professionals found that 52% considered e-cigarettes as ineffective for smoking cessation^5^. In a survey of Chinese general practitioners, 36% of respondents thought that long-term NRT use is unsafe^6^. A majority of United States (US) physicians strongly agreed in a survey that nicotine *per se* contributes to cardiovascular disease (83%) and COPD (81%)^7^. In all these studies, most participants thought that nicotine contributes directly to cancer^4-7^. However, little is known about the situation in tertiary hospitals in Switzerland. This project investigated the knowledge and perceptions of physicians and pharmacists, two healthcare groups mainly responsible for the smoking cessation pharmacotherapy, providing a basis for improving patient care, and increasing cessation rates, which remain low in Switzerland.

## METHODS

This cross-sectional study was conducted in 2023 at the Insel Hospital Group, a tertiary hospital group and one of the biggest healthcare providers in Switzerland. Potential participants, i.e. all physicians and pharmacists of the hospital group, received an invitation to participate voluntarily in the survey via e-mail. The mailing list was provided by the hospital’s human resources department. As pharmacists, unlike physicians, were not recorded by profession, the hospital pharmacy employees and the employees with ‘pharmacist’ in their job title were screened independently by two investigators, and selected on the basis of their job title. The Ethics Committee of the canton Bern reviewed the study and exempted it from approval (Req-2021-01502). The survey was open for four weeks (from 4 April to 1 May 2023) and a reminder was sent after two weeks on 18 April 2023. Participants were informed that all data would be collected and analyzed anonymously without any conclusions being drawn about individual participants.

The survey was conducted using the online survey tool Surveymonkey (SurveyMonkey Inc., San Mateo, California, USA). The questions were developed based on previous studies and the Health Information National Trends Survey (HINTS)^7-13^. The survey domains covered nicotine, smoking cessation, and ENDS knowledge. The questionnaire (the German and French versions used, as well as an English translation) is provided in the Supplementary file. Prior to distribution, the questionnaire was sent to seven healthcare professionals (three physicians and four pharmacists) for pilot testing regarding clarity. The survey included a brief introduction to clarify the terminology used (e.g. difference between snus and nicotine pouches, and different ENDS, e.g. that e-cigarettes use liquid, while heated tobacco products heat tobacco up to 350°C). The ratings on a 0–100 visual analogue scale (VAS) ranged from ‘disagree’ to ‘agree’, ‘not at all harmful’ to ‘very harmful’ or ‘less’ to ‘more’, depending on the question. The middle part corresponded to ‘unsure’, ‘moderately harmful’ or ‘about the same’, respectively. The scale for the question about the efficacy of treatment options ranged from ‘similarly effective’ to ‘five times as effective’. The question on recommendation preferences comprised a discrete scale with the response options: never (0%), rarely (<20%), occasionally (20–50%), often (51–80%), very often (>80%), and always (100%). The final part of the survey asked general and demographic questions, including questions about the participants’ clinical practice and smoking status. Never smokers were defined as having smoked less than 100 tobacco cigarettes in their lifetime. Skipping questions was not an option, but aborting the survey without answering all questions was possible. Participants had the option to choose between German and French as the language.

### Statistical analysis

To achieve a statistical power of 0.9 and a significance level of 0.05, assuming a moderate effect size (0.3), a total of 141 participants would be needed to be recruited, while with the same assumptions but power of 0.8, a total of 107 participants would be needed. The final number of participants depended on the response rate.

All available data were used for the analysis, regardless of whether the participant answered all questions or dropped out of the survey before the end. Categorical variables are presented as frequencies and percentages. Continuous variables are presented as boxplots with Tukey-style whiskers or were grouped into three categories by dividing the VAS lines in three equal parts with the middle section representing uncertainty/about the same, and the sections on the right and on the left representing agreement or disagreement, or ‘more’ or ‘less’ options, depending on the question. For subgroup analysis of categorical variables, a chi-squared test was conducted and for continuous variables, a t-test was conducted. In subgroup analyses the following variables were investigated: gender (men vs women), age (aged ≥41 years vs aged <41 years) and affiliated department (working in pulmonology vs working in other departments, and working in general internal medicine vs working in other departments). Continuous data were tested for normality using the Shapiro-Wilk test. If the data were normally distributed, we performed a one-way repeated-measures ANOVA. *Post hoc* pairwise comparisons were conducted using t-tests with Bonferroni correction. For data that did not conform to normality, we conducted a Friedman test, followed by Wilcoxon signed-rank tests with Bonferroni correction for *post hoc* comparisons. The significance level was set at 0.05. All tests were two-tailed. Data analysis was performed using R (version 4.3.0, R Foundation for Statistical Computing, Vienna, Austria). Data visualization was performed with GraphPad Prism version 8.0.1 (GraphPad Software, La Jolla, California, USA).

## RESULTS

The survey was sent to 2035 healthcare professionals (2009 physicians and 26 pharmacists). Of these, 279 (14%) answered at least one question and 194 (10%) answered all questions. The characteristics of the participants are summarized in Table 1.

**Table 1:** Participants’ characteristics, a cross-sectional study among physicians and pharmacists at a large Swiss hospital group, 2023

*Characteristics*	*n (%)*
**Gender** (N=195)	
Women	103 (53)
Men	91 (47)
Diverse, non-binary or no answer	1 (1)
**Age** (years) (N=195)	
≤30	33 (17)
31–40	102 (52)
41–50	28 (14)
51–60	22 (11)
>60	10 (5)
**Have you ever attended a training on smoking cessation?** (N=203)	
Yes	23 (11)
No	180 (89)
**Department** (N=195)^a^	
General internal medicine	37 (19)
Anesthesiology	26 (13)
Pediatrics	21 (11)
Emergency medicine adults	20 (10)
Neurology	13 (7)
Cardiology	9 (5)
Pulmonology	6 (3)
Radiology	6 (3)
Surgery	5 (3)
Infectiology	5 (3)
Hospital pharmacy	2 (1)
Clinical pharmacy	1 (1)
Other	61 (31)
**Do you smoke tobacco cigarettes?** (N=202)	
Never smoker	155 (77)
Former smoker	33 (16)
Some day smoker	7 (3)
Every day smoker	7 (3)
**Do you regularly (i.e. daily) use electronic nicotine delivery systems (ENDS)?** (N=202)	
Never user	192 (95)
Current user	3 (1)
Former user	7 (3)
**Where do you receive information about ENDS?** (N=196)	
Media	99 (51)
Medical literature	58 (30)
Peers	54 (28)
Patients	21 (11)
Courses for further education	17 (9)
Employees of vape shops	4 (2)
Other sources	3 (2)
Never received any information	71 (36)
**How often do you see patients at your current job** (N=203)	
Daily	173 (85)
Weekly	19 (9)
Less than weekly or never	11 (5)
**Do you ask patients on a regular basis if they smoke? (**N=194)	
Yes	160 (82)
No	34 (18)
**Do you ask patients on a regular basis if they use ENDS?** (N=202)	
Yes	37 (18)
No	165 (82)
**Do you advise patients who smoke to quit smoking on a regular basis?** (N=194)	
Yes	143 (74)
No	51 (26)
**How often do you conduct smoking cessation counseling in your current position?** (N=194)	
Daily	3 (2)
Weekly	24 (12)
Monthly	22 (11)
A few times per year	24 (12)
Once a year or less	13 (7)
Never	108 (56)

a For the affiliated department multiple mentions were possible; departments mentioned by less than 5 participants were grouped as ‘other’, except for pharmacy-related departments.

Participants’ responses regarding the effects of nicotine and whether they are distinct from the effects of other constituents of tobacco smoke, as well as their responses regarding NRT, are presented in Tables 2 and 3, respectively.

**Table 2:** Participants’ answers regarding nicotine, a cross-sectional study among physicians and pharmacists at a large Swiss hospital group, 2023 (N=279)*

*Items*	*n (%)*
**Nicotine is the main substance in tobacco that makes people want to smoke**	
Disagree	25 (9)
Not sure	41 (15)
Agree	213 (76)
**Nicotine on its own causes birth defects**	
Disagree	124 (44)
Not sure	74 (27)
Agree	81 (29)
**Nicotine on its own causes cancer**	
Disagree	204 (73)
Not sure	31 (11)
Agree	44 (16)
**Nicotine on its own causes cardiovascular disease**	
Disagree	114 (41)
Not sure	57 (20)
Agree	108 (39)
**Nicotine on its own causes chronic obstructive pulmonary disease^a,b^**	
Disagree	193 (69)
Not sure	45 (16)
Agree	41 (15)

* No statistical significance detected in subgroup analyses unless stated otherwise.

a Statistical significance in subgroup analysis for respondents working in pulmonology versus other departments (p<0.05).

b Statistical significance in subgroup analysis for women versus men (p<0.001).

**Table 3:** Participants’ answers regarding nicotine replacement therapy, a cross-sectional study among physicians and pharmacists at a large Swiss hospital group, 2023 (N=214, unless stated otherwise)*

*Items*	*n (%)*
**Nicotine replacement products such as nicotine patches and nicotine gum are more or less addictive compared to tobacco cigarettes**	
More	8 (4)
About the same	102 (48)
Less	104 (49)
**Nicotine patches are more or less likely to cause a heart attack than tobacco cigarettes**	
More	0 (0)
About the same	46 (21)
Less	168 (79)
**A cigarette with a lower nicotine content is less or more harmful than a cigarette with a higher nicotine content?^a^ (N=279)**	
More harmful	27 (10)
About the same	220 (79)
Less harmful	32 (11)
**In Switzerland, the cost of nicotine replacement products such as nicotine patches or nicotine gum is currently covered by patient’s basic health insurance^a^**	
True	28 (13)
Not sure	94 (44)
Not true	92 (43)

* No statistical significance detected in subgroup analyses unless stated otherwise.

a Statistical significance in subgroup analysis for respondents working in pulmonology versus other departments (p<0.05).

Most participants disagreed that nicotine on its own causes cancer or COPD (73% and 69%, respectively) and 79% believed that cigarettes with lower nicotine content are about as harmful as cigarettes with higher nicotine content (Table 2). Women were more likely than men to agree that nicotine on its own causes COPD (p<0.001), but no statistically significant differences were found in responses on birth defects, cancer or cardiovascular disease by gender. Participants aged <41 years were no more likely than older participants to agree that nicotine on its own causes birth defects, cancer, cardiovascular disease or COPD. Respondents working in pulmonology compared to respondents from other departments were more likely to disagree that nicotine on its own causes COPD (p<0.05), less likely to respond that a tobacco cigarette with lower nicotine content is less harmful compared to a tobacco cigarette (p<0.05), and more likely to agree that the cost of NRT products is not covered by a patient’s basic health insurance in Switzerland (p<0.05). Otherwise, respondents working in pulmonology or general internal medicine did not respond statistically differently to these questions compared to other specialties.

About half (48%) of the participants thought that NRT products such as nicotine patches and nicotine gums were as addictive as tobacco cigarettes, and most respondents (79%) thought that nicotine patches were less likely to cause a heart attack than tobacco cigarettes (Table 3).

**Figure 1:**
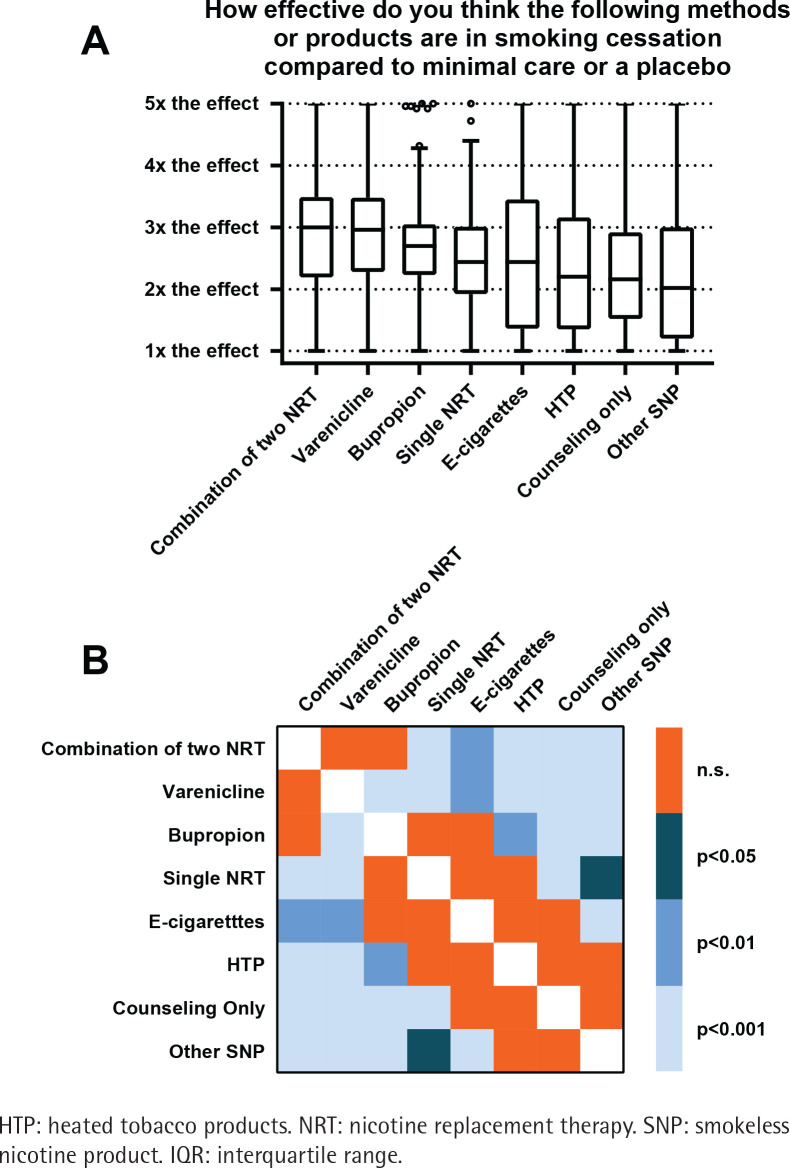
Participants’ estimates of efficacy for various smoking cessation methods, a cross-sectional study among physicians and pharmacists at a large Swiss hospital group, 2023 (N=214): A) Boxplots of participants’ estimates (boxplots span the IQR with a line for the median, whiskers extend to the smallest and largest points within 1.5×IQR); B) Significance plot depicting pairwise statistical comparisons of smoking cessation methods regarding participants’ estimates

**Figure 2:**
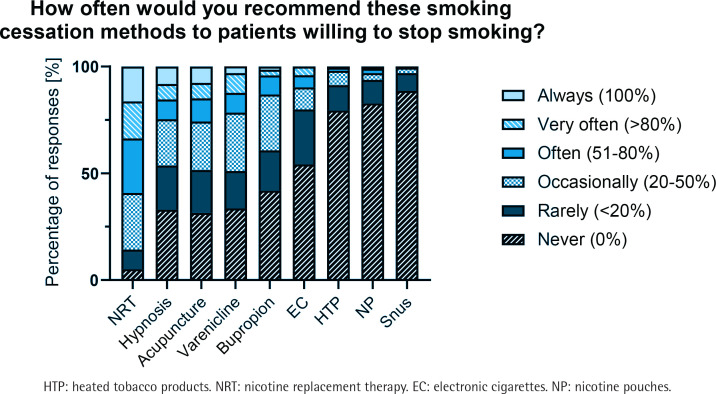
Participants’ answers regarding how often they recommend the various cessation methods to patients willing to quit smoking, a cross-sectional study among physicians and pharmacists at a large Swiss hospital group, 2023 (N=196)

Participants were asked to rate the efficacy of different smoking cessation methods on a VAS scale from 0 to 100, where 0 represents similar efficacy (1×) and 100 represents five times the efficacy (5×) compared to placebo or minimal care, and to indicate how often they would recommend the different methods to patients who want to quit smoking. The results are shown in Figures 1 and 2, respectively.

On average, a combination of two NRT or varenicline were considered the most effective smoking cessation tools, showing a median efficacy estimate three times higher than placebo or minimal care (Figure 1). Bupropion showed a median efficacy estimate of 2.7 times, while e-cigarettes had an efficacy estimate of 2.4 times the effect compared to placebo or minimal care. Counseling only was estimated to be 2.2 more effective than minimal care. When asked how often they would recommend specific smoking cessation products to patients who want to quit smoking, NRT was the most frequent answer with the majority (59%) recommending them at least ‘often (51–80% of the time)’. In contrast, heated tobacco products, nicotine pouches and snus were the least preferred options, with 79%, 83% and 89% of respondents, respectively, stating they would never recommend them (Figure 2).

**Figure 3:**
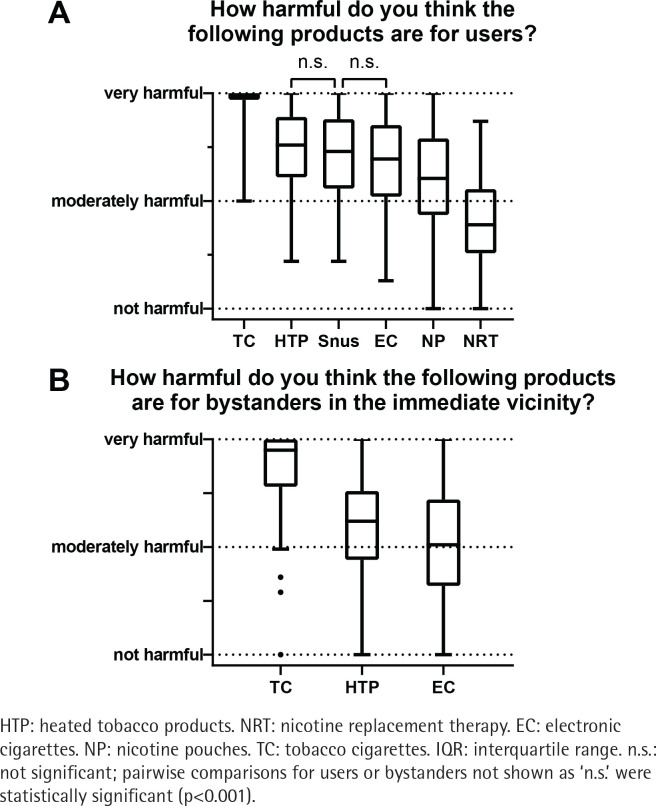
Participants’ answers regarding harm effects of the various products, a cross-sectional study among physicians and pharmacists at a large Swiss hospital group, 2023 (N=203): A) users; B) bystanders in the immediate vicinity (boxplots span the IQR with a line for the median, whiskers extend to the smallest and largest points within 1.5×IQR)

The responses regarding e-cigarettes are shown in Table 4 and the responses regarding the harmful effects of the different products for users and bystanders are shown in Figure 3.

**Table 4:** Participants’ answers regarding e-cigarettes, a cross-sectional study among physicians and pharmacists at a large Swiss hospital group, 2023 (N=203, unless stated otherwise)

*Items*	*n (%)*
**E-cigarettes should be recommended to patients by healthcare professionals as a smoking cessation aid**	
Disagree	128 (63)
Not sure	50 (25)
Agree	25 (12)
**I would recommend e-cigarettes (or recommend them more often) if they were made in pharmaceutical quality**	
Disagree	104 (51)
Not sure	66 (33)
Agree	33 (16)
**E-cigarettes represent a lower or higher public health problem compared to regular tobacco cigarettes**	
Lower	74 (36)
About the same	117 (58)
Higher	12 (6)
**Have you ever advised a patient to visit a vape shop to quit smoking, or would you ever advise them to do so? (N=190)**	
Yes, vape shops should play a role in smoking cessation	5 (3)
Yes, but only if the vape store staff has been trained accordingly	13 (7)
Yes, but only if the person has failed to quit with the first-line approved pharmacotherapies	9 (5)
Yes, for other reasons	2 (1)
No, because that’s the job of healthcare professionals, not vape shops	140 (74)
No, for other reasons	21 (11)

Most participants (63%) opposed healthcare professionals recommending e-cigarettes as a smoking cessation aid, while only 12% supported their use. Additionally, 58% of respondents viewed e-cigarettes as posing a similar public health problem as tobacco cigarettes (Table 4). Tobacco cigarettes were considered the most harmful product for both users and bystanders (Figure 3). Heated tobacco products were considered more harmful than e-cigarettes for both users and bystanders. In contrast, NRT products were viewed as the least harmful product for users. In the *post hoc* analysis, differences between harm perception for users of heated-tobacco products versus snus, and snus versus e-cigarettes, were non-significant. All other pairwise comparisons regarding harmfulness for users or bystanders were statistically significant (p<0.001).

## DISCUSSION

In this anonymous electronic survey conducted mainly among physicians in a large hospital group in Switzerland, most respondents were young women who had never smoked and saw patients daily. About 80% of the respondents said they regularly asked their patients if they smoked (but only a minority asked about ENDS use), about 75% said they regularly advised smokers to quit, and about 25% provided smoking cessation counseling at least once a month. Most participants correctly identified nicotine as the main substance in tobacco that causes addiction and disagreed that nicotine on its own causes cancer or COPD. When asked whether nicotine causes birth defects and cardiovascular disease, responses were more divided. As for newer products such as e-cigarettes, the majority disagreed with recommending them as a smoking cessation aid, while more than half perceived them as equally problematic as tobacco cigarettes for public health.

Difficulty in differentiating the harmful effects of nicotine from those of other harmful components of tobacco smoke might lead to a fear of recommending NRT products, which can be an obstacle to successful smoking cessation. Studies conducted among healthcare professionals in different countries have repeatedly shown that the direct effects of nicotine on health were poorly understood^4-7^. Participants in our study were more likely to correctly identify that nicotine on its own does not cause cancer or COPD. Nicotine is not considered a carcinogenic compound by the International Agency for Research on Cancer (IARC) and nicotine exposure does not increase the risk of COPD when accounting for non-nicotine toxicants^1^. Nicotine is a sympathomimetic substance that can increase heart rate and cardiac contractility, but there is little evidence that nicotine on its own causes cardiovascular disease. However, it may contribute to acute cardiovascular events in pre-existing cardiovascular disease^14^. In Switzerland, NRT in pregnancy is generally only encouraged as a measure of last resort^15^. Concerns have been raised primarily because nicotine crosses the placental barrier and accumulates in the fetus^16^, and animal studies have shown that maternal nicotine use has adverse effects on the newborn^17^. However, systematic reviews have not found an association between NRT use during pregnancy and adverse birth outcomes^18^. Notably, the potential risks associated with NRT use during pregnancy should be weighed against the definitive risks of continuing smoking^19^.

Two studies conducted in the US by the same research group found that physicians identifying as women were more likely than their male counterparts to agree that nicotine directly contributes to birth defects and cancer^7,20^. No such association was found in our study, but women were more likely to agree that nicotine on its own causes COPD. Pulmonologists were previously reported to be more likely to disagree that nicotine causes COPD^7^, which we also found in our study. Adequate knowledge of nicotine and smoking cessation is fundamental for all healthcare professionals, although some specialties (e.g. pulmonology, general internal medicine) may be more likely to be confronted with this topic. Generally, respondents may have linked nicotine to addiction and, consequently, to smoking-related health effects. The wording ‘Nicotine *on its own* causes …’ was used to emphasize that nicotine’s direct effects were asked, as this phrasing has been shown to reduce alleged misperceptions^20^.

Most participants correctly identified that cigarettes with lower nicotine content are not less harmful than cigarettes with higher nicotine content. As smokers titrate their daily consumption to maintain desired nicotine levels, switching from high to low nicotine cigarettes could result in more intense smoking behavior or to smoking more cigarettes per day, which exposes users to more harmful constituents^21,22^. False beliefs that lower nicotine content cigarettes might be less harmful have been found in surveys among the US adult population^10^. While lowering the nicotine content might lead to lower abuse liability of a product, it might also contribute to the false impression that such products are harmless or less harmful than other cigarettes, making smokers less willing to quit^23^.

The fundamental principle of tobacco harm reduction is that substituting high-risk products (e.g. tobacco cigarettes) with lower risk products (e.g. e-cigarettes) reduces harm. In a US survey study, more than half of the participating physicians believed that all tobacco products are equally harmful^24^. Participants in our study considered tobacco cigarettes to be significantly more harmful than any other product, recognizing the reduced harm potential of e-cigarettes, snus, or nicotine pouches, for example. In our survey, heated tobacco products were rated as less harmful than tobacco cigarettes but more harmful than e-cigarettes. While e-cigarettes are currently considered less harmful than tobacco cigarettes^25^, there is insufficient evidence to support the use of heated tobacco products as harm reduction tools, as they have not been proven to produce fewer deleterious effects than smoking, even though they may reduce exposure to harmful substances^26^. This point is supported by the FDA’s refusal in 2020 to consider heated tobacco as a reduced-risk product^27^. Participants considered NRT to be the least harmful, but some participants still considered it harmful, which may make them reluctant to prescribe NRT.

The use of NRT is supported by a well-established and favorable safety profile, even when used beyond the recommended 12-week period, and they effectively help smokers quit^28^, with better efficacy when combining two NRT products (patch as long-acting and one short-acting product)^29^. However, their costs are generally not covered by the health insurance in Switzerland (with some rare exceptions in case of supplementary insurance options). Varenicline and bupropion are the other currently approved first-line treatments for smoking cessation besides NRT, while the evidence base for other interventions such as hypnosis is still insufficient^30^. Cytisine, a nicotinic α4β2 receptor partial agonist, has been shown to be effective for smoking cessation, but is not available on the Swiss market and was therefore not included in our survey^31^. In general, participants slightly overestimated the effectiveness of smoking cessation interventions. Participants estimated that varenicline triples smoking cessation rates compared to placebo, but the relative risk is estimated at 2.32 (95% CI: 2.15–2.51)^31^. The same trend was observed for all products.

Healthcare professionals seem to oppose e-cigarettes as a tool in smoking cessation therapy, with only about 10% agreeing that these devices should be recommended for this indication. However, there is high-certainty evidence that e-cigarettes can increase quit rates compared to NRT^32^. Furthermore, e-cigarettes are used as smoking cessation aids among recent ex-smokers in Switzerland^33^. Regarding risks for bystanders, e-cigarettes do not generate smoke and their aerosol emissions have a much shorter duration/effect (lifetime 10–20 s) than the secondhand smoke of conventional cigarettes (approximately 1.4 h)^34^. E-cigarettes are thought to be a less harmful alternative to tobacco cigarettes; however, they are not without risks. One of the drawbacks for recommending e-cigarettes is that the market is insufficiently regulated and health concerns have been raised about certain ingredients such as the various flavorings that might contain potential inhalation toxicants^35^. However, the quality of e-cigarettes did not seem to be a major concern among the participants when it comes to recommending them, as only a small minority agreed that they would recommend e-cigarettes (or recommend them more often) if they were produced in pharmaceutical quality. Correspondingly, healthcare professionals were reluctant to include vape shops in smoking cessation therapies, even though according to a survey of vape shop managers in Switzerland most of them had received referrals from physicians^36^. The recommendations given by the vape shop managers to smokers in this previous survey varied widely and only a minority of the managers had attended a smoking cessation course^36^. If a future project were to involve vape stores more actively in smoking cessation efforts, it should include evidence-based, nationally or internationally coordinated recommendations.

### Strengths and limitations

Limitations of this study include the cross-sectional design, which does not allow for causality statements and the very small number of participating pharmacists compared to physicians The low response rate limits the generalizability of the results and the statistical power to detect significant differences in subgroups of interest. The questions were based on similar previous projects and were pilot-tested before starting; however, the questionnaire was not validated and only covered some aspects of nicotine and smoking cessation. Participation was voluntary and based on self-reports, which may introduce sampling and response bias, and some might have consulted the internet for the correct answer. Further limitations include the lack of adjusted analyses to account for potential confounders, the single-center design, which may limit the generalizability to other hospitals within Switzerland and to other countries. For questions assessing the harmfulness of products, subjective terminology was used, so that absolute statements of these responses should be made with caution. On the other hand, to our knowledge, this is the first survey to investigate health professionals’ beliefs about the health risks of tobacco smoking, nicotine, and smoking cessation products conducted in a large urban hospital group in Switzerland.

## CONCLUSIONS

This survey identified knowledge gaps regarding the properties of nicotine and smoking cessation therapy while also exploring health professionals’ attitudes towards newer products such as e-cigarettes. It is necessary that medical providers discuss smoking habits with all their patients and provide adequate advice. Based on the knowledge gaps identified, specific measures such as targeted information and teaching sessions can be implemented. Given that more than half of the participants relied on the media for information, it is important to emphasize the role of the media in providing accurate and reliable content. For example, by clarifying the misperception that nicotine can cause cancer, healthcare professionals may be more inclined to encourage patients to use NRT products to facilitate smoking cessation. Future work could further investigate attitudes and misperceptions in other groups of healthcare professionals, and re-evaluate their knowledge and practices following a cycle of training on this topic.

## Supplementary Material



## Data Availability

The data supporting this research are available from the authors on reasonable request.
