# Supplementary material for: Knowledge and perceptions of nicotine, smoking cessation and electronic nicotine delivery systems among physicians and pharmacists in a Swiss hospital group
Source: Tob Induc Dis. 2025 Jul 24;23:10.18332/tid/204839. doi: 10.18332/tid/204839 (PMC12288835; doi:10.18332/tid/204839)
Supplement: Supplementary file 1 [file TID-23-103-s1.pdf]

KNOWLEDGE AND PERCEPTION REGARIDING NICOTINE, SMOKING CESSATION, AND ELECTRONIC NICOTINE DELIVERY SYSTEMS WITHIN THE HOSPITAL GROUP

NICOTINE

Would you agree or disagree with the following statements? Please move the slider in the direction, for which answer seems most probable/correct to you. **The slider must be moved to submit an answer.**

1. Nicotine is the main substance in tobacco that makes people want to smoke.

Fully disagree — Not sure — Fully agree

2. Nicotine on its own causes cancer.

Fully disagree — Not sure — Fully agree

3. Nicotine on its own causes birth defects.

Fully disagree — Not sure — Fully agree

4. Nicotine on its own causes cardiovascular disease.

Fully disagree — Not sure — Fully agree

5. Nicotine on its own causes chronic obstructive pulmonary disease (COPD).

Fully disagree — Not sure — Fully agree

6. Do you think that a cigarette with a lower nicotine content is less or more harmful than a cigarette with a higher nicotine content?

Less harmful | About the same | More harmful

SMOKING CESSATION

Information on the different terms and products:

Alternative nicotine products allow users to take in nicotine without the combustion of tobacco and the inhalation of the harmful substances produced by it. These include electronic devices (Electronic Nicotine Delivery Systems, "ENDS"), such as ***e-cigarettes (also known as vapes or vaporizers)****, which heat a usually nicotine-containing liquid, and* ***heated tobacco products (e.g. IQOS, Ploom, Glo, also known as "heat-not-burn" products)****, which heat tobacco up to 350°C. Also included are* ***snus (oral nicotine pouches WITH tobacco)*** *and* ***nicotine pouches (oral nicotine pouches WITHOUT tobacco)****.*

Evidence-based pharmacotherapies include ***nicotine replacement therapy products*** *such as nicotine patches, nicotine gum, nicotine inhalers, nicotine spray, and nicotine lozenges, as well as* ***varenicline (Champix®)****, a partial agonist at the nicotinic acetylcholine α4β2 receptor, and* ***bupropion (Zyban®)****, a norepinephrine and dopamine reuptake inhibitor.*

1. How effective do you think the following methods or products are compared to minimal care or placebo for smoking cessation?

Medical counselling alone

Similar efficacy | Three-times the efficacy (3x) | Five-times the efficacy (5x)

Bupropion (Zyban®)

Similar efficacy | Three-times the efficacy (3x) | Five-times the efficacy (5x)

Varenicline (Champix®)

Similar efficacy | Three-times the efficacy (3x) | Five-times the efficacy (5x)

Single nicotine replacement therapy product (e.g. nicotine patch)

Similar efficacy | Three-times the efficacy (3x) | Five-times the efficacy (5x)

Combination of two nicotine replacement products (e.g. nicotine patch and nicotine gum)

Similar efficacy | Three-times the efficacy (3x) | Five-times the efficacy (5x)

E-cigarettes (vapes)

Similar efficacy | Three-times the efficacy (3x) | Five-times the efficacy (5x)

Heated tobacco products

Similar efficacy | Three-times the efficacy (3x) | Five-times the efficacy (5x)

Other smokeless nicotine products (e.g. snus, nicotine pouches)

Similar efficacy | Three-times the efficacy (3x) | Five-times the efficacy (5x)

2. Do you think that nicotine replacement products such as nicotine patches and nicotine gums are more or less likely to lead to addiction compared to tobacco cigarettes?

less | about the same | more

3. Do you think that nicotine patches are more or less likely to cause a heart attack than cigarettes?

less | about the same | more

4. In Switzerland, the costs of nicotine replacement products such as nicotine patches or nicotine gums are currently covered by the patient's basic health insurance.

☐ True ☐ Not true ☐ I am not sure

ELECTRONIC NICOTINE DELIVERY SYSTEMS (ENDS)

Would you agree or disagree with the following statement?

1. E-cigarettes (vapes) should be recommended to patients by a medical professional as a smoking cessation tool.

Fully disagree — Not sure — Fully agree

2. I would recommend e-cigarettes (or recommend them more often), if they were made in pharmaceutical quality.

Fully disagree — Not sure — Fully agree

3. In your opinion, how harmful are the following products for users?

Tobacco cigarettes

Not harmful — Moderately harmful — Very harmful

E-cigarettes (vapes)

Not harmful — Moderately harmful — Very harmful

Heated tobacco products

Not harmful | Moderately harmful | Very harmful

Snus

Not harmful | Moderately harmful | Very harmful

Nicotine Pouches

Not harmful | Moderately harmful | Very harmful

Nicotine replacement products (e.g. nicotine patch, nicotine gum, nicotine inhaler)

Not harmful | Moderately harmful | Very harmful

4. In your opinion, how harmful are the following products for bystanders in the immediate vicinity?

   Tobacco cigarettes (second-hand smoke)

Not harmful | Moderately harmful | Very harmful

   E-cigarettes (passive inhalation of vapour)

Not harmful | Moderately harmful | Very harmful

Heated tobacco products (second-hand emissions)

Not harmful | Moderately harmful | Very harmful

5. Compared to combustible cigarettes, do you think that e-cigarettes represent a lower or higher public health problem?

Much lower | About the same | Much higher

Do you have any additions/comments to the main questions? ___________________________

GENERAL AND DEMOGRAPHIC DATA

How often do you see patients in your current position?

☐ daily
☐ weekly
☐ monthly
☐ less than monthly
☐ never

Do you regularly ask your patients if they smoke?

☐ Yes
☐ No

Do you regularly advise your patients who smoke to quit smoking?

☐ Yes
☐ No

In your current position, how often do you conduct smoking cessation counselling?

☐ daily
☐ weekly
☐ monthly
☐ a couple of times a year
☐ less than once yearly
☐ never

Have you ever attended a training course on smoking cessation? If yes, which one?

☐ Yes ____________________
☐ No

Do you smoke tobacco cigarettes?

☐ I have never smoked (< 100 cigarettes or < 5 packs in my life)
☐ I am a former smoker (at least 100 cigarettes or 5 packs in my life but I have quit now)
    Former smoker: How many cigarettes did you use to smoke on an average day?
☐ I smoke on some days
    On how many of the last 30 days did you smoke cigarettes?
    On the days you smoked in the last 30 days, how many cigarettes did you smoke on average per day?
☐ I smoke every day
    Daily smoker: How many cigarettes do you usually smoke on an average day?

Have you ever regularly (i.e. daily) used electronic nicotine delivery systems (ENDS), such as e-cigarettes or heated tobacco? If "former user" or "current user", please indicate which product(s) (multiple answers possible)

☐ I have never been a regular ENDS user
☐ Former user ☐ e-cigarettes ☐ heated tobacco product
☐ other: __________
☐ Current user ☐ e-cigarettes ☐ heated tobacco product
☐ other: __________

Has a patient ever asked you about ENDS? If "yes" please indicate about which product(s) (multiple answers possible)

☐ Yes ☐ e-cigarettes ☐ heated tobacco product
☐ other: __________
☐ No

Do you regularly ask your patients if they use ENDS? → if "yes", please indicate which product(s) (multiple answers possible)

☐ Yes ☐ e-cigarettes ☐ heated tobacco
☐ other: __________

☐ No

How often do you recommend the following smoking cessation products to your patients who want to quit smoking?

	Never (0%)	Rarely (<20%)	Occasionally (20-50%)	Often (51-80%)	Very often (>80%)	Always (100%)
Nicotine replacement therapy (e.g. nicotine patch, nicotine gum)	☐	☐	☐	☐	☐	☐
Varenicline (Champix®)	☐	☐	☐	☐	☐	☐
Buproprion (Zyban®)	☐	☐	☐	☐	☐	☐
E-cigarettes (vapes)	☐	☐	☐	☐	☐	☐
Heated tobacco products	☐	☐	☐	☐	☐	☐
Snus	☐	☐	☐	☐	☐	☐
Nicotine pouches	☐	☐	☐	☐	☐	☐
Acupuncture	☐	☐	☐	☐	☐	☐
Hypnosis	☐	☐	☐	☐	☐	☐

Have you ever advised a patient to visit a vape shop to quit smoking, or would you ever advise them to do so?

☐ No, because that is the job of health care professionals and not vape shops
☐ Yes, but only if the vape shop staff received proper training
☐ Yes, but only if patients have failed to quit with the approved first-line pharmacotherapies (nicotine replacement therapy, varenicline (Champix®), bupropion (Zyban®))
☐ Yes, vape shops should play a role in smoking cessation therapy
☐ Other: ______________________________

Where do you get information about ENDS such as e-cigarettes and heated tobacco from?

☐ press
☐ medical literature
☐ colleagues
☐ patients
☐ training course
☐ vape shop staff
☐ until now, no information received
☐ other: _______________

What is your gender?

Male	Female	Diverse, non-binary or no answer
☐	☐	☐

How old are you [years]?

< 25	25-30	31-35	36-40	41-45	46-50	51-55	56-60	> 60
☐	☐	☐	☐	☐	☐	☐	☐	☐

In which department do you work? (multiple answers possible)

☐ Allergology	☐ Heart surgery	☐ Osteoporosis
☐ Angiology	☐ Hospital pharmacy	☐ Otorhinolaryngology
☐ Anaesthesiology	☐ Human Genetics	☐ Paediatrics
☐ Cardiology	☐ Infectiology	☐ Plastic and hand surgery
☐ Clinical Chemistry	☐ Intensive Care Medicine	☐ Pulmonology
☐ Clinical Pharmacy	☐ Medical Oncology	☐ Proctology
☐ Dermatology	☐ Nephrology	☐ Psychooncology
☐ Diabetology and Endocrinology	☐ Neurology	☐ Radiology
☐ Emergency medicine for adults	☐ Neuroradiology	☐ Radio-Oncology
☐ Emergency medicine for children and adolescents	☐ Neurosurgery	☐ Rehabilitation
☐ General internal medicine	☐ Nuclear Medicine	☐ Rheumatology and Immunology
☐ Gastroenterology	☐ Nutritional Medicine and Metabolism	☐ Surgery
☐ Gynaecology	☐ Oncology	☐ Thoracic surgery
☐ Geriatrics	☐ Ophthalmology	☐ Urology
☐ Haematology and Central Haematology Laboratory	☐ Oral and maxillofacial surgery	☐ Vascular surgery
☐ Hand surgery	☐ Orthopaedic surgery and traumatology	☐ Visceral surgery

Do you have any additions/comments to the general questions and demographic data part?

____

WISSEN UND WAHRNEHMUNG IN BEZUG AUF NIKOTIN, RAUCHENTWOÖHNUNG UND ELEKTRONISCHE NIKOTINABGABESYSTEME IN DER INSEL GRUPPE

NIKOTIN

Würden Sie den folgenden Aussagen zustimmen oder nicht? Bitte bewegen Sie den Schieberegler in die Richtung, in welcher die Antwort Ihnen am wahrscheinlichsten/korrektesten scheint. **Der Schieberegler muss bewegt werden, um eine Antwort abzugeben.**

1. Nikotin ist der Hauptinhaltsstoff in Tabak, der die Personen zum Rauchen verleitet.

Stimme überhaupt nicht zu — Nicht sicher — Stimme vollständig zu

2. Nikotin für sich allein verursacht Krebs.

Stimme überhaupt nicht zu — Nicht sicher — Stimme vollständig zu

3. Nikotin für sich allein verursacht Geburtsfehler.

Stimme überhaupt nicht zu — Nicht sicher — Stimme vollständig zu

4. Nikotin für sich allein verursacht Herz-Kreislauf-Erkrankungen.

Stimme überhaupt nicht zu — Nicht sicher — Stimme vollständig zu

5. Nikotin für sich allein verursacht chronisch obstruktive Lungenerkrankung (COPD).

Stimme überhaupt nicht zu — Nicht sicher — Stimme vollständig zu

6. Denken Sie, dass eine Zigarette mit einem niedrigen Nikotingehalt weniger oder mehr schädlich ist als eine Zigarette mit einem höheren Nikotingehalt?

Viel weniger schädlich | Ungefähr gleich | Viel schädlicher

RAUCHENTWÖHNUNG

Information zu den verschiedenen Begriffen und Produkten:

Durch alternative Nikotinprodukte können Benutzende Nikotin aufnehmen, ohne Tabakverbrennung und Inhalation der dadurch erzeugten schädlichen Stoffe. Dazu gehören elektronische Geräte (electronic nicotine delivery systems, „ENDS“) wie die ***E-Zigaretten (auch E-Dampfer oder Vaporiser genannt)****, welche eine meistens nikotinhaltige Flüssigkeit erhitzen, und die* ***Tabakerhitzer (z.B IQOS, Ploom, Glo, auch „Heat-not-Burn“ Produkte genannt)****, die Tabak auf bis zu 350°C erhitzen, aber auch* ***Snus (Nikotinbeutel zum oralen Gebrauch MIT Tabak)*** *und* ***„Nicotine Pouches“ (Nikotinbeutel zum oralen Gebrauch OHNE Tabak)****.*

Zu den evidenzbasierten Pharmakotherapien gehören die ***Nikotinersatztprodukte*** *wie Nikotinpflaster, Nikotinkaugummi, Nikotininhalator, Nikotinspray und Nikotinlutschtabletten,* ***Vareniclin (Champix®)****, ein partieller Agonist am nikotinergen Acetylcholin α4β2 Rezeptor, und der Noradrenalin und Dopamin Wiederaufnahmehemmer* ***Bupropion (Zyban®).***

1. Wie wirksam sind Ihrer Meinung nach die folgenden Methoden oder Produkte im Vergleich zu Minimalversorgung oder einem Placebo, um mit dem Rauchen aufzuhören?

Nur medizinische Beratung

Ähnliche Wirkung | Verdreifachung der Wirkung (3x) | Verfünffachung der Wirkung (5x)

Bupropion (Zyban®)

Ähnliche Wirkung | Verdreifachung der Wirkung (3x) | Verfünffachung der Wirkung (5x)

Vareniclin (Champix®)

Ähnliche Wirkung | Verdreifachung der Wirkung (3x) | Verfünffachung der Wirkung (5x)

Einzelnes Nikotinersatzprodukt (z.B. Nikotinpflaster)

Ähnliche Wirkung | Verdreifachung der Wirkung (3x) | Verfünffachung der Wirkung (5x)

Kombination von zwei Nikotinersatzprodukten (z.B. Nikotinpflaster und Nikotinkaugummi)

Ähnliche Wirkung | Verdreifachung der Wirkung (3x) | Verfünffachung der Wirkung (5x)

E-Zigaretten (E-Dampfer)

Ähnliche Wirkung | Verdreifachung der Wirkung (3x) | Verfünffachung der Wirkung (5x)

Tabakerhitzer

Ähnliche Wirkung | Verdreifachung der Wirkung (3x) | Verfünffachung der Wirkung (5x)

Andere rauchfreie Nikotinprodukte (z.B. Snus, Nicotine Pouches)

Ähnliche Wirkung | Verdreifachung der Wirkung (3x) | Verfünffachung der Wirkung (5x)

2. Denken Sie, dass Nikotinersatzprodukte wie Nikotinpflaster und Nikotinkaugummis im Vergleich zu Tabakzigaretten mehr oder weniger zur Abhängigkeit führen?

weniger | ungefähr gleich | mehr

3. Denken Sie, dass Nikotinpflaster mehr oder weniger wahrscheinlich sind, einen Herzinfarkt zu verursachen als Tabakzigaretten?

weniger — ungefähr gleich — mehr

4. In der Schweiz werden die Kosten für Nikotinersatzprodukte wie Nikotinpflaster oder Nikotinkaugummis derzeit von der Grundversicherung der PatientInnen übernommen.

☐ Stimmt ☐ Stimmt nicht ☐ Ich bin nicht sicher

ELEKTRONISCHE NIKOTINABGABESYSTEME (ENDS)

Würden Sie den folgenden Aussagen zustimmen oder nicht?

1. E-Zigaretten (E-Dampfer) sollten von einer medizinischen Fachperson als Hilfsmittel zur Rauchentwöhnung an PatientInnen empfohlen werden.

Stimme überhaupt nicht zu — Nicht sicher — Stimme vollständig zu

2. Ich würde E-Zigaretten (E-Dampfer) empfehlen (oder sie öfter empfehlen), wenn sie in pharmazeutischer Qualität hergestellt werden würden.

Stimme überhaupt nicht zu — Nicht sicher — Stimme vollständig zu

3. Wie schädlich sind Ihrer Meinung nach die folgenden Produkte für die Benutzenden?

Tabakzigaretten

Überhaupt nicht schädlich | Mässig schädlich | Sehr schädlich

E-Zigaretten (E-Dampfer)

Überhaupt nicht schädlich | Mässig schädlich | Sehr schädlich

Tabakerhitzer

Überhaupt nicht schädlich | Mässig schädlich | Sehr schädlich

Snus

Überhaupt nicht schädlich | Mässig schädlich | Sehr schädlich

Nicotine Pouches

Überhaupt nicht schädlich | Mässig schädlich | Sehr schädlich

Nikotinersatzprodukte (z.B. Pflaster, Kaugummi, Inhalator)

Überhaupt nicht schädlich | Mässig schädlich | Sehr schädlich

4. Wie schädlich sind Ihrer Meinung nach die folgenden Produkte für unbeteiligte Personen in unmittelbarer Nähe?

   Tabakzigaretten (Passivrauchen)

   Überhaupt nicht schädlich — Mässig schädlich — Sehr schädlich

   E-Zigaretten (passive Inhalation von Dampf)

   Überhaupt nicht schädlich — Mässig schädlich — Sehr schädlich

   Tabakerhitzer (passive Inhalation von Emissionen)

   Überhaupt nicht schädlich — Mässig schädlich — Sehr schädlich

5. Sind Sie der Meinung, dass E-Zigaretten (E-Dampfer) im Vergleich zu gewöhnlichen Tabakzigaretten ein geringeres oder grösseres Problem für die öffentliche Gesundheit darstellen?

   Viel geringer — Ungefähr gleich — Viel grösser

Haben Sie Ergänzungen/Bemerkungen zu den Hauptfragen? ________________________

ALLGEMEINE UND DEMOGRAFISCHE DATEN

Wie oft sehen Sie an Ihrer derzeitigen Arbeitsstelle PatientInnen?

☐ täglich
☐ wöchentlich
☐ monatlich
☐ weniger als monatlich
☐ nie

Fragen Sie Ihre PatientInnen regelmässig, ob sie rauchen?

☐ Ja
☐ Nein

Raten Sie regelmässig Ihren rauchenden PatientInnen, mit dem Rauchen aufzuhören?

☐ Ja
☐ Nein

Wie oft führen Sie bei Ihrer derzeitigen Arbeitsstelle Rauchstoppberatungen durch?

☐ täglich
☐ wöchentlich
☐ monatlich
☐ ein paar Mal im Jahr
☐ einmal pro Jahr oder weniger
☐ nie

Haben Sie jemals an einer Schulung zur Rauchentwöhnung teilgenommen? Wenn ja, an welcher?

☐ Yes ________________________
☐ No

Rauchen Sie Tabakzigaretten?

☐ Ich habe nie geraucht (< 100 Zigaretten oder < 5 Packungen in meinem Leben)
☐ Ich bin ein/e ehemalige/r RaucherIn (mindestens 100 Zigaretten oder 5 Packungen in meinem Leben, aber ich habe jetzt aufgehört)
    Wie viele Zigaretten haben Sie an einem durchschnittlichen Tag geraucht?
☐ Ich rauche an manchen Tagen
    An wie vielen der letzten 30 Tage haben Sie Zigaretten geraucht?
    An den Tagen, an denen Sie in den letzten 30 Tagen geraucht haben, wie viele Zigaretten haben Sie im Durchschnitt pro Tag geraucht?
☐ Ich rauche jeden Tag
    Wie viele Zigaretten rauchen Sie normalerweise an einem durchschnittlichen Tag?

Haben Sie jemals regelmässig (d.h. täglich) elektronische Nikotinabgabesysteme (ENDS) wie E-Zigaretten oder Tabakerhitzer verwendet? Falls «ehemalige/r NutzerIn» oder «aktuelle/r NutzerIn», geben Sie bitte an, welche(s) Produkt(e) (Mehrfachnennung möglich)

☐ Ich war nie ein/e regelmässige/r ENDS-NutzerIn
☐ Ehemalige/r NutzerIn ☐ E-Zigaretten (E-Dampfer) ☐ Tabakerhitzer
☐ Anderes: __________
☐ Aktuelle/r NutzerIn ☐ E-Zigaretten (E-Dampfer) ☐ Tabakerhitzer
☐ Anderes: __________

Hat Sie jemals ein/e PatientIn nach ENDS gefragt? → Wenn «ja», geben Sie bitte an, über welche(s) Produkt(e) (Mehrfachnennung möglich)

☐ Ja ☐ E-Zigaretten (E-Dampfer) ☐ Tabakerhitzer
☐ Anderes: __________
☐ Nein

Fragen Sie regelmässig Ihre PaitentInnen, ob sie ENDS benutzen? → Wenn “ja”, geben Sie bitte an, welche(s) Produkt(e) (Mehrfachnennung möglich)

☐ Ja ☐ E-Zigaretten (E-Dampfer) ☐ Tabakerhitzer
☐ Anderes: __________
☐ Nein

Wie oft würden Sie PatientInnen, die mit dem Rauchen aufhören möchten, die folgenden Produkte oder Methoden zur Rauchentwöhnung empfehlen?

	Nie (0%)	Selten (<20%)	Gelegentlich (20-50%)	Häufig (51-80%)	Sehr häufig (>80%)	Immer (100%)
Nikotinersatztherapie (z.B. Nikotinpflaster, Nikotinkaugummi)	☐	☐	☐	☐	☐	☐
Vareniclin (Champix®)	☐	☐	☐	☐	☐	☐
Buproprion (Zyban®)	☐	☐	☐	☐	☐	☐
E-Zigaretten (E-Dampfer)	☐	☐	☐	☐	☐	☐
Tabakerhitzer	☐	☐	☐	☐	☐	☐
Snus	☐	☐	☐	☐	☐	☐
Nicotine pouches	☐	☐	☐	☐	☐	☐
Akupunktur	☐	☐	☐	☐	☐	☐
Hypnose	☐	☐	☐	☐	☐	☐

Haben Sie jemals einem/r PatientIn geraten, einen Vapeshop aufzusuchen, um mit dem Rauchen aufzuhören, oder würden Sie jemals dazu raten, dies zu tun?

☐ Nein, weil das die Aufgabe von Fachleuten im Gesundheitswesen und nicht von Vapeshops ist.
☐ Ja aber nur, wenn das Personal der Vapeshops entsprechend geschult wurde.
☐ Ja, aber nur, wenn die Person mit den zugelassenen Erstlinien-Pharmakotherapien (Nikotinersatzpräparate, Vareniclin (Champix®), Bupropion (Zyban®)) nicht aufhören konnte.
☐ Ja, Vapeshops sollten eine Rolle in der Rauchentwöhnung spielen.
☐ Anderes: ________________________________

Woher erhalten Sie Informationen über ENDS wie E-Zigaretten oder Tabakerhitzer? (Mehrfachnennung möglich)

☐ Presse
☐ Medizinische Fachliteratur
☐ KollegInnen
☐ PatientInnen
☐ Fortbildungskurs
☐ Mitarbeitende von Vapeshops
☐ Bis jetzt keine Informationen erhalten
☐ Sonstiges: ________________

Was ist Ihr Geschlecht?

Männlich	Weiblich	Divers, nicht-binär oder keine Antwort
☐	☐	☐

Wie alt sind Sie? [Jahre]

< 25	25-30	31-35	36-40	41-45	46-50	51-55	56-60	> 60
☐	☐	☐	☐	☐	☐	☐	☐	☐

In welcher Abteilung arbeiten Sie? (Mehrfachnennung möglich)

☐ Allergologie	☐ Humangenetik	☐ Nuklearmedizin
☐ Allgemeine Innere Medizin	☐ Hämatologie und Hämatologisches Zentrallabor	☐ Onkologie
☐ Angiologie	☐ Infektiologie	☐ Orthopädische Chirurgie und Traumatologie
☐ Anästhesiologie	☐ Intensivmedizin	☐ Osteoporose
☐ Augenheilkunde	☐ Kardiologie	☐ Plastische- und Handchirurgie
☐ Chirurgie	☐ Kinderheilkunde	☐ Pneumologie
☐ Dermatologie	☐ Klinische Chemie	☐ Proktologie
☐ Diabetologie und Endokrinologie	☐ Klinische Pharmazie	☐ Psychoonkologie
☐ Ernährungsmedizin und Metabolismus	☐ Medizinische Onkologie	☐ Radiologie
☐ Frauenheilkunde	☐ Schädel-, Kiefer- und Gesichtschirurgie	☐ Radio-Onkologie
☐ Gastroenterologie	☐ Nephrologie	☐ Rehabilitation
☐ Gefässchirurgie	☐ Neurochirurgie	☐ Rheumatologie und Immunologie
☐ Geriatrie	☐ Neurologie	☐ Spitalpharmazie
☐ Hals- Nasen-, und Ohrenkrankheiten	☐ Neuroradiologie	☐ Thoraxchirurgie
☐ Handchirurgie	☐ Notfallmedizin Erwachsene	☐ Urologie
☐ Herzchirurgie	☐ Notfallmedizin für Kinder und Jugendliche	☐ Viszeralchirurgie

Haben Sie Ergänzungen/Anmerkungen zu den allgemeinen Fragen und den demografischen Daten?

____

CONAISSANCES ET PERCEPTIONS DE LA NICOTINE, DU SEVRAGEE TABAGIQUE ET DES SYSTÈMES ÉLECTRONIQUES D'ADMINISTRATION DE NICOTINE DANS LE GROUPE HOSPITALIER DE L'INSEL

NICOTINE

Êtes-vous d'accord ou non avec les affirmations suivantes? Veuillez déplacer le curseur dans la direction où la réponse vous semble la plus probable/correcte. **Le curseur doit être déplacé pour donner une réponse.**

1. La nicotine est la principale substance contenue dans le tabac qui donne envie de fumer.

Pas du tout d'accord | Pas sûr | Tout à fait d'accord

2. La nicotine, à elle seule, provoque le cancer.

Pas du tout d'accord | Pas sûr | Tout à fait d'accord

3. La nicotine, à elle seule, provoque des malformations congénitales.

Pas du tout d'accord | Pas sûr | Tout à fait d'accord

4. La nicotine, à elle seule, provoque des maladies cardiovasculaires.

Pas du tout d'accord | Pas sûr | Tout à fait d'accord

5. La nicotine, à elle seule, provoque une bronchopneumopathie chronique obstructive (BPCO).

Pas du tout d'accord | Pas sûr | Tout à fait d'accord

6. Pensez-vous qu'une cigarette à faible teneur en nicotine est moins ou plus nocive qu'une cigarette à forte teneur en nicotine?

Beaucoup moins nocive | À peu près pareil | Plus nocive

SEVRAGE TABAGIQUE

Informations sur les différents termes et produits:

Les produits alternatifs à base de nicotine permettent aux utilisateurs d'absorber la nicotine sans brûler le tabac et sans inhaler les substances nocives qui en résultent. Il s'agit de dispositifs électroniques (electronic nicotine delivery systems, « ENDS » tels que la ***cigarette électronique (aussi appelée vaporette ou vaporisateur)*** *qui chauffe un liquide contenant généralement de la nicotine, ainsi que* ***les produits de tabac chauffé (par ex. IQOS, Ploom, Glo, aussi appelées « Heat-not-Burn »)*** *qui chauffent le tabac jusqu'à 350°C, mais aussi le* ***snus (sachets de nicotine à usage oral AVEC tabac)*** *et les* ***« Nicotine Pouches » (sachets de nicotine à usage oral SANS tabac).***

Les pharmacothérapies fondées sur des données probantes incluent les ***substituts nicotiniques*** *tels que les patchs à la nicotine, les gommes à la nicotine, l'inhalateur de nicotine, le spray de nicotine et les comprimés à sucer à la nicotine, la* ***varénicline (Champix®)****, un agoniste partiel du récepteur nicotinique de l'acétylcholine α4β2, et le* ***bupropion (Zyban®),*** *un inhibiteur de la recapture de la noradrénaline et de la dopamine.*

1. Selon vous, quelle est l'efficacité des méthodes ou produits suivants par rapport à des soins minimaux ou à un placebo pour arrêter de fumer ?

Conseil médical uniquement

Efficacité similaire | Efficacité triplée (x3) | Efficacité quintuplée (x5)

Bupropion (Zyban®)

Efficacité similaire | Efficacité triplée (x3) | Efficacité quintuplée (x5)

Varénicline (Champix®)

Efficacité similaire | Efficacité triplée (x3) | Efficacité quintuplée (x5)

Un seul produit de substitution nicotinique (par ex. patch de nicotine)

Efficacité similaire | Efficacité triplée (x3) | Efficacité quintuplée (x5)

Combinaison de deux substituts nicotiniques (par ex patch de nicotine et gomme à la nicotine)

Efficacité similaire | Efficacité triplée (x3) | Efficacité quintuplée (x5)

Cigarettes électroniques (vaporettes)

Efficacité similaire | Efficacité triplée (x3) | Efficacité quintuplée (x5)

Produits du tabac chauffés

Efficacité similaire | Efficacité triplée (x3) | Efficacité quintuplée (x5)

Produits à base de nicotine sans fumée (par ex. snus, sachets de nicotine)

Efficacité similaire | Efficacité triplée (x3) | Efficacité quintuplée (x5)

2. En comparaison avec les cigarettes combustibles, pensez-vous que les substituts nicotiniques tels que les patchs et les gommes à la nicotine sont plus ou moins susceptibles d'entraîner und dépendance?

moins | à peu près pareil | plus

3. Pensez-vous que les patchs à la nicotine sont plus ou moins susceptibles de provoquer une crise cardiaque que les cigarettes?

moins | à peu près pareil | plus

4. En Suisse, les coûts des substituts nicotiniques tels que les patchs ou les gommes à la nicotine sont actuellement couverts par l'assurance maladie de base du patient.

☐ Vrai ☐ Faux ☐ Je ne suis pas sûr

SYSTÈMES ÉLECTRONIQUES DE DÉLIVRANCE DE LA NICOTINE (ENDS)

Êtes-vous d'accord ou non avec les affirmations suivantes?

1. Les cigarettes électroniques (vaporettes) comme outil de sevrage tabagique devraient être recommandées aux patients par un professionnel de santé.

Pas du tout d'accord | Pas sûr | Tout à fait d'accord

2. Je recommanderais les cigarettes électroniques (vaporettes) (ou les recommanderais plus souvent), si elles étaient fabriquées en qualité pharmaceutique.

Pas du tout d'accord | Pas sûr | Tout à fait d'accord

3. À votre avis, dans quelle mesure les produits suivants sont-ils nocifs pour les utilisateurs?

Cigarettes combustibles

Pas du tout nocif | Modérément nocif | Très nocif

Cigarettes électroniques (vaporettes)

Pas du tout nocif | Modérément nocif | Très nocif

Produits de tabac chauffés

Pas du tout nocif | Modérément nocif | Très nocif

Snus

Pas du tout nocif | Modérément nocif | Très nocif

Sachets de nicotine

Pas du tout nocif | Modérément nocif | Très nocif

Substituts nicotiniques (par ex. patch, gomme, inhalateur)

Pas du tout nocif | Modérément nocif | Très nocif

4. À votre avis, dans quelle mesure les produits suivants sont-ils nocifs pour les personnes non impliquées qui se trouvent dans les environs immédiats?

   Cigarettes combustibles (tabagisme passif)

   Pas du tout nocif | Modérément nocif | Très nocif

   Cigarettes électroniques (inhalation passive de la vapeur)

   Pas du tout nocif | Modérément nocif | Très nocif

   Produits de tabac chauffés (inhalation passive d'émissions)

   Pas du tout nocif | Modérément nocif | Très nocif

5. Pensez-vous que les cigarettes électroniques (vaporettes) représentent un problème de santé publique de gravité inférieure ou supérieure par rapport aux cigarettes de tabac ordinaires?

   Gravité inférieure | À peu près pareil | Gravité supérieure

Avez-vous des ajouts/commentaires aux questions principales? ________________________

DONÉES GÉNÉRALES ET DÉMOGRAPHIQUES

À quelle fréquence voyez-vous des patients dans votre lieu de travail actuel?

☐ Fréquence quotidienne
☐ Fréquence hebdomadaire
☐ Fréquence mensuelle
☐ Moins d'une fois par mois
☐ Jamais

Demandez-vous régulièrement à vos patients s'ils fument?

☐ Oui
☐ Non

Conseillez-vous régulièrement à vos patients qui fument d'arrêter de fumer ?

☐ Oui
☐ Non

Dans votre travail actuel, à quelle fréquence conduisez-vous des consultations pour arrêter de fumer?

☐ Fréquence quotidienne
☐ Fréquence hebdomadaire
☐ Fréquence mensuelle
☐ Quelques fois par an
☐ Une fois par an ou moins
☐ Jamais

Avez-vous déjà participé à une formation sur le sevrage tabagique? Si oui, laquelle?

☐ Oui ________________________
☐ Non

Fumez-vous des cigarettes de tabac?

☐ Je n’ai jamais fumé (c’est-à-dire < 100 cigarettes ou < 5 paquets dans ma vie).
☐ Je suis un(e) ancien(ne) fumeur(se) (au moins 100 cigarettes ou 5 paquets dans ma vie mais j’ai arrêté maintenant).
Combien de cigarettes fumiez-vous en moyenne par jour?
☐ Je fume certains jours.
Au cours des 30 derniers jours, à combien de jours avez-vous fumé?
Les jours où vous avez fumé au cours des 30 derniers jours, combien de cigarettes avez-vous fumé en moyenne par jour?
☐ Je fume tous les jours.
Combien de cigarettes fumez-vous habituellement sur une journée typique?

Avez-vous déjà utilisé régulièrement (c.-à-d. tous les jours) des systèmes électroniques de délivrance de nicotine (ENDS), tels que les cigarettes électroniques ou le tabac chauffé ?

☐ Je n’ai jamais été un(e) utilisateur(trice) régulier(e) d’ENDS
☐ Ancien(ne) utilisateur(trice) ☐ Cigarettes électroniques ☐ Tabac chauffé
☐ Autre: __________
☐ Utilisateur(trice) actuel(le) ☐ Cigarettes électroniques ☐ Tabac chauffé
☐ Autre: __________

Un(e) patient(e) vous a-t-il/elle déjà posé des questions sur ENDS? Si « oui », indiquez à propos de quel(s) produit(s) sur la page suivante s’il vous plaît

☐ Oui ☐ Cigarettes électroniques ☐ Tabac chauffé
☐ Autre: __________
☐ Non

Demandez-vous régulièrement à vos patients s’ils utilisent l’ENDS? Si “oui”, indiquez quel(s) produit(s) sur la page suivante s’il vous plaît.

☐ Oui ☐ Cigarettes électroniques ☐ Tabac chauffé
☐ Autre: __________
☐ Non

Avec quelle fréquence recommanderiez-vous aux patients(es) qui souhaitent arrêter de fumer les produits ou méthodes suivants pour le sevrage tabagique?

	Jamais (0%)	Rarement (<20%)	Occasionnellement (20-50%)	Souvent (51-80%)	Très souvent (>80%)	Toujours (100%)
Thérapie de remplacement de la nicotine (par ex. patch de nicotine, gommes à la nicotine)	☐	☐	☐	☐	☐	☐
Varénicline (Champix®)	☐	☐	☐	☐	☐	☐
Buproprion (Zyban®)	☐	☐	☐	☐	☐	☐
Cigarettes électroniques (Vaporettes)	☐	☐	☐	☐	☐	☐
Tabac chauffé	☐	☐	☐	☐	☐	☐
Snus	☐	☐	☐	☐	☐	☐
Sachets de nicotine	☐	☐	☐	☐	☐	☐
Acupuncture	☐	☐	☐	☐	☐	☐
Hypnose	☐	☐	☐	☐	☐	☐

Avez-vous déjà conseillé à un(e) patient(e) de visiter un magasin de cigarettes électroniques pour arrêter de fumer, ou conseilleriez-vous leur de le faire?

☐ Non, parce que l'accompagnement du sevrage tabagique incombe aux professionnels de santé et non pas aux magasins de cigarettes électroniques.
☐ Oui mais seulement si le personnel des magasins de cigarettes électroniques recevait une formation adéquate au préalable.
☐ Oui mais seulement si la personne n'a pas réussi à arrêter de fumer avec les pharmacothérapies approuvées de première intention (substituts nicotiniques, varénicline (Champix®), bupropion (Zyban®)).
☐ Oui, les magasins de cigarettes électroniques devraient jouer un rôle dans la thérapie de sevrage tabagique.
☐ Autre: ________________________________

D'où obtenez-vous des informations sur les ENDS comme les cigarettes électroniques et le tabac chauffé? (plusieurs réponses possibles)

☐ La presse
☐ Littérature médicale
☐ Collègues
☐ Patient(e)s
☐ Cours de formation
☐ Employés de magasins de cigarettes électroniques
☐ Jusqu'à présent, aucune information reçue
☐ Autres: ________________

Quel est votre sexe?

Homme	Femme	Divers, non-binaire ou pas de réponse
☐	☐	☐

Quel âge avez-vous [ans]?

< 25	25-30	31-35	36-40	41-45	46-50	51-55	56-60	> 60
☐	☐	☐	☐	☐	☐	☐	☐	☐

Dans quel département travaillez-vous? (plusieurs réponses possibles)

☐ Allergologie	☐ Médecine génétique	☐ Médecine nucléaire
☐ Médecine interne générale	☐ Hématologie et laboratoire hématologie central	☐ Oncologie
☐ Angiologie	☐ Infectiologie	☐ Chirurgie orthopédique et traumatologie
☐ Anesthésiologie	☐ Médecine intensive	☐ Ostéoporose
☐ Ophtalmologie	☐ Cardiologie	☐ Chirurgie de la main et plastique
☐ Chirurgie	☐ Pédiatrie	☐ Pneumologie
☐ Dermatologie	☐ Médecine de laboratoire	☐ Proctologie
☐ Diabétologie et Endocrinologie	☐ Pharmacie clinique	☐ Psycho-oncologie
☐ Médecine alimentaire et métabolisme	☐ Oncologie médicale	☐ Radiologie
☐ Gynécologie	☐ Chirurgie crânio-maxillo faciale	☐ Radio-oncologie
☐ Gastroentérologie	☐ Néphrologie	☐ Réhabilitation
☐ Chirurgie vasculaire	☐ Neurochirurgie	☐ Rhumatologie et immunologie
☐ Gériatrie	☐ Neurologie	☐ Pharmacie hospitalière
☐ Oto-rhino-laryngologie	☐ Neuroradiologie	☐ Chirurgie thoracique
☐ Chirurgie de la main	☐ Médecine d’urgence adultes	☐ Urologie
☐ Chirurgie cardiaque	☐ Médecine d’urgence enfants et jeunes	☐ Chirurgie viscérale

Avez-vous des ajouts/commentaires à faire sur la partie questions générales et données démographiques?

____

© 2025 Christen S.E. et al.
